# A surgical Decision-making scoring model for spontaneous ventilation- and mechanical ventilation-video-assisted thoracoscopic surgery in non-small-cell lung cancer patients

**DOI:** 10.1186/s12893-023-02150-z

**Published:** 2023-09-25

**Authors:** Runchen Wang, Qixia Wang, Hengrui Liang, Zhiming Ye, Jiawen Qiu, Yu Jiang, Jianxing He, Lei Zhao, Wei Wang

**Affiliations:** 1 Department of Thoracic Surgery and Oncology, The First Affiliated Hospital of Guangzhou Medical University, State Key Laboratory of Respiratory Disease, National Clinical Research Center for Respiratory Disease, Guangzhou Institute of Respiratory Health, Guangzhou, China; 2 Department of Physiology, School of Basic Medical Sciences, Guangzhou Medical University, Guangzhou, 511495 China

**Keywords:** Non-small cell lung cancer, Spontaneous ventilation-video-assisted thoracoscopic surgery, Mechanical ventilation-video-assisted thoracoscopic surgery, Surgical decision-making scoring, Nomogram

## Abstract

**Backgrounds:**

Spontaneous ventilation-video-assisted thoracoscopic surgery (SV-VATS) has been applied to non-small cell lung cancer (NSCLC) patients in many centers. Since it remains a new and challenging surgical technique, only selected patients can be performed SV-VATS. We aim to conduct a retrospective single-center study to develop a clinical decision-making model to make surgery decision between SV-VATS and MV (mechanical ventilation) -VATS in NSCLC patients more objectively and individually.

**Methods:**

Four thousand three hundred sixty-eight NSCLC patients undergoing SV-VATS or MV-VATS in the department of thoracic surgery between 2011 and 2018 were included. Univariate and multivariate regression analysis were used to identify potential factors influencing the surgical decisions. Factors with statistical significance were selected for constructing the Surgical Decision-making Scoring (SDS) model. The performance of the model was validated by area under the receiver operating characteristic curve (AUC), calibration curves and decision curve analysis (DCA).

**Results:**

The Surgical Decision-making Scoring (SDS) model was built guided by the clinical judgment and statistically significant results of univariate and multivariate regression analyses of potential predictors, including smoking status (*p* = 0.03), BMI (*p* < 0.001), ACCI (*p* = 0.04), T stage (*p* < 0.001), N stage (*p* < 0.001), ASA grade (*p* < 0.001) and surgical technique (*p* < 0.001). The AUC of the training group and the testing group were 0.72 and 0.70, respectively. The calibration curves and the DCA curve revealed that the SDS model has a desired performance in predicting the surgical decision.

**Conclusions:**

This SDS model is the first clinical decision-making model developed for an individual NSCLC patient to make decision between SV-VATS and MV-VATS.

**Supplementary Information:**

The online version contains supplementary material available at 10.1186/s12893-023-02150-z.

## Background

Lung cancer is one of the most common epithelial tumors with a high rate of morbidity and mortality [1], about 85% of which are non-small cell lung cancer (NSCLC) [2]. Radical surgery plays a critical role in the systemic strategy of treatment for operable NSCLC. The traditional open thoracotomy is recommended as golden procedure to conduct radical surgery for operable NSCLC [3]. With the promotion of the concept of enhanced recovery after surgery, the video-assisted thoracoscopic surgery (VATS) has been recommended to perform radical surgery for early-stage NSCLC currently [4, 5]. In recent years, with the continuous updating of thoracoscopic instruments, equipment and the increasingly sophisticated surgical skills of surgeons, many thoracic centers have tried to carry out spontaneous ventilation VATS (SV-VATS) in NSCLC patients [6–9]. Evidence has showed that it is safe and feasible to employ SV-VATS in NSCLC patients and the effect of SV-VATS was comparable to mechanical ventilation (MV)-VATS [3, 10]. SV-VATS avoids the tracheal injury caused by endotracheal intubation [6]. Besides, by reducing the use of neuromuscular blocking agents and opioid analgesia during surgery, SV-VATS has a lower risk of postoperative respiratory failure, postoperative hyperalgesia, and even opioid dependence [11]. Moreover, growing evidence has suggested that the SV-VATS can accelerate postoperative recovery, reduce complications, hospital stay, and medical costs compared with MV-VATS [12]. However, SV-VATS imposes higher requirements on proficient technical skills and quick decision-making skills of the surgical team. Besides, it is still lack of SV-VATS consensus or guideline for NSCLC patients at present. It remains uncertain what kind of NSCLC patients can undergo SV-VATS, which restrict more extensive application of SV-VATS in NSCLC patients to some extent. Indeed, selecting proper patients is the first step to success in a surgery. Therefore, we aim to quantify the factors influencing surgery selection for NSCLC patients by constructing a surgical decision-making scoring (SDS) model (a pre-operative model), to find out the certain NSCLC patients that can undergo SV-VATS based on clinical characteristics.

## Methods

### IRB information

The study was performed based on the data from the first affiliated hospital of Guangzhou Medical University, the institutional review boards at which approved the retrospective analysis of anonymous data and waived the need to obtain patient informed consent (IRB Report ID: 2018-57).

### Study population selection

**Fig. 1:**
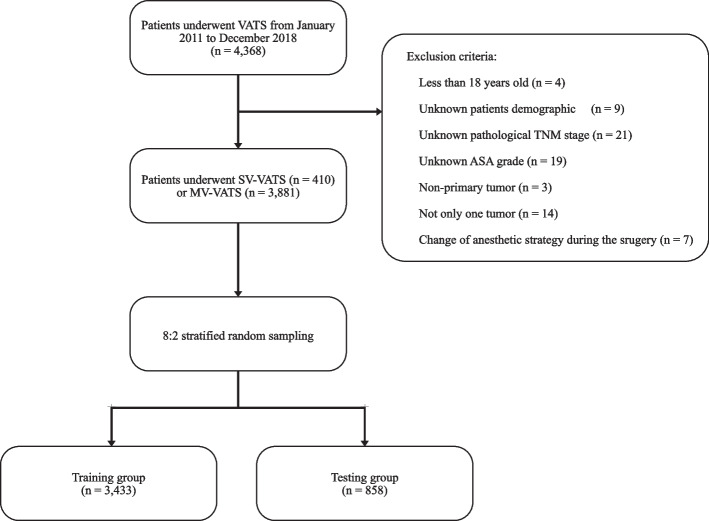
Flowchart of inclusion and exclusion process. VATS: video-assisted thoracoscopic surgery; SV: spontaneous ventilation; MV: mechanical ventilation; T: tumor; N: node; M: metastasis; ASA: American society of anesthesiologists

Four thousand three hundred sixty-eight NSCLC patients undergoing SV-VATS or MV-VATS in the department of thoracic surgery of the first affiliated hospital of Guangzhou Medical University between 2011 and 2018 were identified. Exclusion criteria are as follows: (1) Less than 18 years old; (2) Unknown patients demographic; (3) Unknown pathological TNM stage; (4) Unknown American society of anesthesiologists (ASA) grade; (5) Non-primary tumor; (6) Not only one tumor; (7) Change of anesthetic strategy during the surgery. All data were anonymously extracted in a double-blinded manner. Patient demographic characteristics included age, body mass index (BMI), smoking status, gender, age-adjusted Charlson comorbidity index (ACCI), and forced expiratory volume in 1 s (FEV1)/forced volume vital capacity (FVC) ratio after inhalation of bronchodilators. After exclusions, 4,291 patients met the inclusion criteria were included in this study (Fig. 1). Oncological information, such as tumor location, pathological T stage, N stage, and M stage were included. The tumor pathological stage was categorized based on the 8th TNM guideline stipulated by American Joint Committee on Cancer [13]. Operation information included American society of anesthesiologists (ASA) grade and surgical technique. Surgical technique includes lung segmentectomy and lobectomy, both of which are anatomical surgical resection. Informed consent was obtained from each of the patients after explaining the reason, modalities, risks, and benefits of the surgery. Senior doctors will provide a recommendation for the surgical approach based on a thorough assessment of the patient’s individual circumstances and the principle of maximizing patient benefit. Informed consent was obtained from each of the patients after explaining the reason, modalities, risks, and benefits of the surgery. The final decision of the surgical type (MV-VATS or SV-VATS) was made jointly by the thoracic surgeons, anesthetists, and patients before the operation.

### SV-VATS technique

Dexmedetomidine (1.0 mg/kg/h for 15 min), target-controlled infusion (TCI) of propofol (2–3.5 mg/mL), and intravenous infusion sufentanil (0.2 mg/kg) were used for anesthesia induction. The third-generation double-tube LMA was used for ventilation management. If the patients have no spontaneous ventilation, manual ventilation or simultaneous intermittent mandatory ventilation mode will be used to assist ventilation during anesthesia induction. A bispectral index (BIS) sensor was used for evaluation of sedation level.

During the anesthesia maintenance period, intercostal incision local anesthesia, visceral pleural surface anesthesia, and vagus nerve block were performed with lidocaine or ropivacaine in SV-VATS to decrease the use of remifentanil, maintaining spontaneous breathing. TCI of propofol, remifentanil, and dexmedetomidine were administered at 1.5 to 4 mg/mL, 0.03 to 0.08 mg/kg/min, and 0.5 to 1.0 mg/kg/h, respectively. BIS was maintained between 45 and 60 during the operation. Dexmedetomidine was stopped directly after the pleural cavity closure, and propofol and remifentanil were stopped at the end of the operation. The anesthetic was not inhaled during the procedure. The procedure of SV-VATS technique was shown in Supplementary Fig. 1.

### Statistical analysis

Patients were randomly divided into a training group and a testing group at a ratio of 8:2. To evaluate the balance and difference between the two groups, categorical variables presented as frequencies and percentages were compared using the Chi-square test or Fisher’s exact test, while continuous variables were compared by Student’s t-test or Mann-Whitney U test. Univariate logistic regression analysis was used to identify potential factors influencing the surgical decisions. Factors with statistical significance by univariate regression analysis were further entered into multivariate regression analysis. Odds ratio (OR) values and 95% confidence intervals (CI) calculations were included in multivariate regression analysis. The variables that were statistically significant (*p* < 0.05) in the multivariate analysis were selected for constructing the SDS model.

### Model validation

To assess the performance of the model, three metrics were employed: discrimination, calibration, and clinical usefulness. Discrimination assesses the ability of the model to differentiate between patients undergoing SV-VATS and MV-VATS. The discrimination was evaluated using the area under the receiver operating characteristic curve (AUC). Values of AUC range from 0.5 and 1.0, with 0.5 indicating no discrimination, greater than 0.7 indicating a reasonable estimate, and 1.0 indicating perfect discrimination [14]. A calibration curve was drawn to evaluate the calibration, which analyzed the agreement between the observed and estimated outcomes. In addition, decision curve analysis (DCA) was applied to evaluate the clinical usefulness of the model [15].

### Software

All statistical analysis was performed using R 4.0.5 (The R Core Team, R Foundation for Statistical Computing, Vienna, Austria) running on R Studio 1.4.1106 (R Studio Team, R Studio Inc. Boston, MA, USA) with packages: foreign [16], regplot [17], ggprism [18], rms [19], pROC [20], ggDCA [21], and do [22]. Statistical significance was set at 2-sided *p* < 0.05.

## Results

### Study population characteristics

Demographic characteristics, oncological information, and operation information of the patients in the SV-VATS and MV-VATS groups were shown Table 1, 410 patients underwent the SV-VATS and 3,881 patients underwent the MV-VATS. All patients who underwent the SV-VATS or MV-VATS received the PS score assessment before the surgery. And all patients included has a PS score between 0 and 1. The information of training group and testing group were shown in Table 2. After an 8:2 stratified random sampling, 3,433 patients were included in the training group, 858 patients were included in the testing group. No significant difference was observed between the training group and testing group.

**Table 1:** Summary statistics demographic information in the SV-VATS group and the MV-VATS group

Variables	SV-VATS		MV-VATS	
	Number	SD	Number	SD
Age		11.45		11.17
60	192		1201	
60–75	186		2092	
75	32		588	
Gender		0.50		0.49
Male	190		2256	
Female	220		1625	
Smoking status		0.42		0.48
Smoking	93		1462	
Nonsmoking	317		2419	
BMI		2.35		2.94
18.5	23		214	
18.5–24	306		2310	
24	81		1357	
ACCI		1.23		1.15
0	27		100	
1	65		288	
2	102		814	
3	130		1329	
4	73		1031	
5	13		319	
T stage^a^		0.63		0.78
1	305		2171	
2	87		1266	
3	9		310	
4	9		134	
N stage^a^		0.65		0.84
0	347		2735	
1	20		290	
2	41		839	
3	2		17	
M stage^a^		0.28		0.29
0	376		3518	
1	34		363	
FEV1/FVC^b^		0.35		0.41
70% (1)	352		3030	
≥ 70% (0)	58		851	
ASA grade		0.23		0.27
1	12		45	
2	389		3598	
3	9		238	
Surgical technique		0.85		0.53
Lung segmentectomy	97		301	
Lobectomy	313		3580	

*SV-VATS* Spontaneous ventilation video-assisted thoracoscopic surgery, *MV-VATS* Mechanical ventilation video-assisted thoracoscopic surgery, *SD* Standard deviation, *BMI* Body mass index, *ACCI* Age-adjusted Charlson Comorbidity Index, *T* Tumor, *N* Node, *M* Metastasis, *FEV1* Forced expiratory volume in one second, *FVC* Forced vital capacity, *ASA* American society of anesthesiologists

^a^Pathological stages based on the 8^th^ edition of the American Joint Committee on Cancer (AJCC)

^b^FEV1/FVC after inhalation of bronchodilators

**Table 2:** Summary statistics demographic information in the training set and the test group

Variables	Training group		Testing group		*p*-Value
	Number	SD	Number	SD	
Age		11.32		11.13	0.82
60	1116		277		
60–75	1817		461		
75	500		120		
Gender		0.49		0.50	0.41
Male	1968		478		
Female	1465		380		
Smoking status		0.48		0.48	0.29
Smoking	1258		297		
Nonsmoking	2175		561		
BMI		2.89		2.93	0.53
18.5	188		49		
18.5–24	2090		809		
24	1155		283		
ACCI		1.17		1.17	0.91
0	102		25		
1	286		67		
2	730		186		
3	1160		299		
4	894		210		
5	261		71		
T stage^a^		0.77		0.86	0.74
1	1970		506		
2	1096		257		
3	254		65		
4	113		30		
N stage^a^		0.83		0.84	0.20
0	2458		624		
1	262		48		
2	697		183		
3	16		3		
M stage^a^		0.29		0.28	0.61
0	3111		783		
1	322		75		
FEV1/FVC^b^		0.41		0.40	0.69
70%	2701		681		
≥ 70%	732		177		
ASA grade		0.26		0.28	0.64
1	44		13		
2	3196		791		
3	193		54		
Surgical technique		0.57		0.61	0.10
Lung segmentectomy	306		92		
Lobectomy	3127		766		

*SD* Standard deviation, *BMI* Body mass index, *ACCI* Age-adjusted Charlson Comorbidity Index, *T* Tumor, *N* Node, *M* Metastasis, *FEV1* Forced expiratory volume in one second, *FVC* Forced vital capacity, *ASA* American society of anesthesiologists

^a^Pathological stages based on the 8^th^ edition of the American Joint Committee on Cancer (AJCC)

^b^FEV1/FVC after inhalation of bronchodilators

### Identify factors independently associated with surgical decision

Univariate and multivariate logistic regression analyses were applied to identify independent factors affecting surgical decision.

In the univariate regression analysis, age (Odds ratio (OR) = 0.97, 95% confidence interval (CI) = 0.96–0.98, *P* < 0.001), gender (OR = 0.57, 95% CI = 0.45–0.73, *P* < 0.001), smoking status (OR = 0.49, 95% CI = 0.37–0.66, *P* < 0.001), BMI (OR = 0.90, 95% CI = 0.87–0.95, *P* < 0.001), ACCI (OR = 0.75, 95% CI = 0.67–0.83, *P* < 0.001), T stage (OR = 0.59, 95% CI = 0.49–0.70, *P* < 0.001), N stage (OR = 0.63, 95% CI = 0.52–0.75, *P* < 0.001), FEV1/FVC after inhalation of bronchodilators (OR = 1.77, 95% CI = 1.27–2.55, *P* = 0.001), ASA grade (OR = 0.42, 95% CI = 0.24–0.72, *P* = 0.002) and surgical technique (OR = 0.53, 95% CI = 0.46–0.62, *P* < 0.001) were identified to be significant factors associated with the surgical decision (Table 3).

**Table 3:** Summary of the results the univariate logistic regression

Variables	Odds ratio	95% CI	*p*-Value
Age	0.97	0.96–0.98	< 0.001
Gender	0.57	0.45–0.73	< 0.001
Smoking status	0.49	0.37–0.66	< 0.001
BMI	0.90	0.87–0.95	< 0.001
ACCI	0.75	0.67–0.83	< 0.001
T stage^a^	0.59	0.49–0.70	< 0.001
N stage^a^	0.63	0.52–0.75	< 0.001
M stage^a^	0.80	0.50–1.24	0.35
FEV1/FVC^b^	1.77	1.27–2.55	0.001
Tumor location	1.04	0.99–1.09	0.06
ASA grade	0.42	0.24–0.72	0.002
Surgical technique	0.53	0.46–0.62	< 0.001

*CI* Confidence interval, *BMI* Body mass index, *ACCI* Age-adjusted Charlson Comorbidity Index, *T* Tumor, *N* Node, *M* Metastasis, *FEV1* Forced expiratory volume in one second, *FVC* Forced vital capacity, *ASA* American society of anesthesiologists

^a^Pathological stages based on the 8^th^ edition of the American Joint Committee on Cancer (AJCC)

^b^FEV1/FVC after inhalation of bronchodilators

The factors with statistical significance in univariate analysis were included as variables in further multivariate regression analysis. The results of multivariate regression analysis showed that smoking status (OR = 0.69, 95% CI = 0.49–0.96, *P* = 0.03), BMI (OR = 0.87, 95% CI = 0.84–0.91, *P* < 0.001), ACCI (OR = 0.76, 95% CI = 0.59–0.98, *P* = 0.04), T stage (OR = 0.70, 95% CI = 0.58–0.83, *P* < 0.001), N stage (OR = 0.70, 95% CI = 0.58–0.84, *P* < 0.001), ASA grade (OR = 0.51, 95% CI = 0.30–0.85, *P* < 0.001) and surgical technique (OR = 0.61, 95% CI = 0.53–0.72, *P* < 0.001) were independent factors for surgical decision (Table 4).

**Table 4:** Summary of the results the multivariate logistic regression

Variables	Odds ratio	95% CI	*p*-Value
Age	1.00	0.98–1.03	0.93
Gender	0.99	0.74–1.32	0.93
Smoking status	0.69	0.49–0.96	0.03
BMI	0.87	0.84–0.91	< 0.001
ACCI	0.76	0.59–0.98	0.04
T stage^a^	0.70	0.58–0.83	< 0.001
N stage^a^	0.70	0.58–0.84	< 0.001
FEV1/FVC^b^	1.13	0.81–1.61	0.48
ASA grade	0.51	0.30–0.85	< 0.001
Surgical technique	0.61	0.53–0.72	< 0.001

*CI* Confidence interval, *BMI* Body mass index, *ACCI* Age-adjusted Charlson Comorbidity Index, *T* Tumor, *N* Node, *FEV1* Forced expiratory volume in one second, *FVC* Forced vital capacity, *ASA* American society of anesthesiologists

^a^Pathological stages based on the 8^th^ edition of the American Joint Committee on Cancer (AJCC)

^b^FEV1/FVC after inhalation of bronchodilators

### Development of an individualized prediction model

**Fig. 2:**
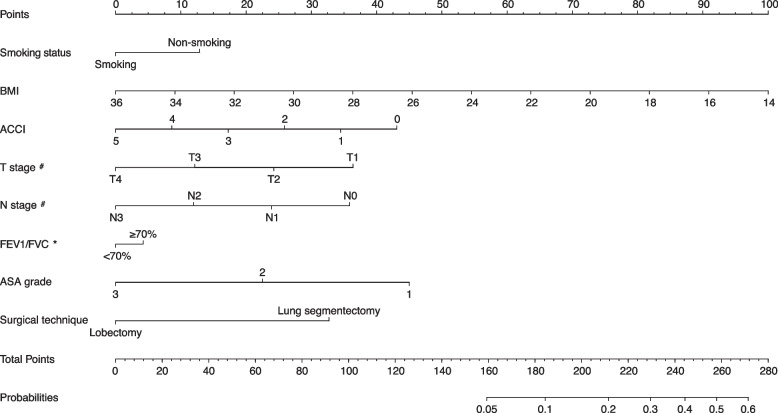
Nomogram for decision making between SV-VATS and MV-VATS in NSCLC patients. SV-VATS: spontaneous ventilation video-assisted thoracoscopic surgery; MV-VATS: mechanical ventilation video-assisted thoracoscopic surgery; BMI: body mass index; ACCI: age-adjusted Charlson Comorbidity Index; T: tumor; N: node; FEV1: forced expiratory volume in 1 s; FVC: forced volume vital capacity; ^#^: pathological stages based on the 8^th^ edition of the American Joint Committee on Cancer (AJCC); *: FEV1/FVC after inhalation of bronchodilators

The SDS model was built guided by the clinical judgment and statistically significant results of univariate and multivariate regression analyses of potential predictors. Finally, smoking status, BMI, ACCI, T stage, N stage, FEV1/FVC after inhalation of bronchodilators, ASA grade, and surgical technique were included in the model. The Nomogram of SDS model was displayed in Fig. 2. Each variable in the nomogram was assigned a point. The total points could be used to confer the possibility for further SV-VATS.

### Model validation and clinical application

**Fig. 3:**
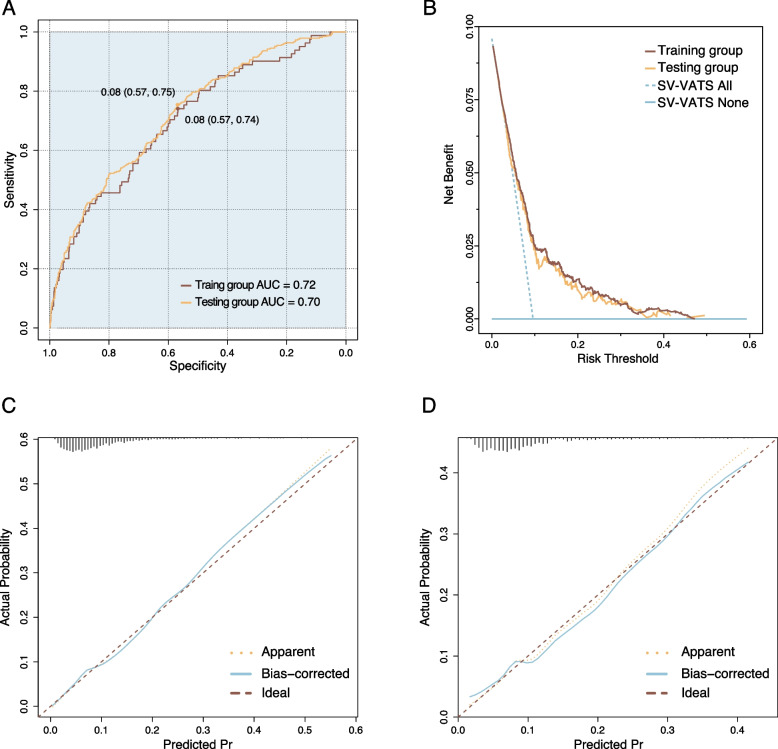
**A** Receiver operating characteristic (ROC) curves for surgical decision-making scoring (SDS) model; **B** Decision curve analysis of SDS model; **C** Calibration curves for SDS model in training group; **D** Calibration curves for SDS model in testing group

The SDS model showed desired performance in predicting the decision of the SV-VATS. Respectively, the AUC of the training group and the testing group were 0.72 (0.08, 0.57–0.74) and 0.70 (0.08, 0.57–0.75) (Fig. 3A).

DCA curve showed that the SDS model were better than the “all treatment” and “no treatment” indexes in the training set and testing set (Fig. 3B). Moreover, the calibration curve of the training group and testing group showed the surgical decision-making scoring (SDS) model had a satisfactory consistency and high calibration degree in prediction of the patients who will benefit from the SV-VATS (Fig. 3C, D).

We have also prepared a comprehensive guide for clinicians and other readers on the practical implementation of the SDS model in a clinical setting (supplementary material).

## Discussion

With the development of SV-VATS, the safety, feasibility, and advantages of this new surgical mode have been recognized by more and more thoracic surgeons.

Although it remains a new and challenging surgical technique, several studies from different centers in recent years have proved the safety and feasibility in NSCLC patients underwent SV-VATS compared MV-VATS. Zheng et al. proved that invasive NSCLC patients undergoing SV-VATS lobectomy have better long-term outcomes compared with MV-VATS [23]. Xu et al. proved that the intraoperative bleeding in SV-VATS is less than MV-VATS, while the operating time is not significantly different between SV-VATS and MV-VATS. Patients showed satisfaction to SV-VATS for its advantages in reducing postoperative complications and accelerating postoperative recovery compared to MV-VATS [6]. One possible reason may be that SV-VATS attenuate the inflammatory responses caused by surgery and stimulate cellular immune function. SV-VATS reduced patient subjective discomfort after surgery [24, 25]. Without tracheal intubation, SV-VATS reduces the adverse effects such as intubation-related airway trauma, residual neuromuscular blockade and irritable, and postoperative cough [26]. Besides, SV-VATS reduces the risk of moderate or more thoracic effusion and is associated with shorter extubating time [6, 27]. Shorter extubating time is associated with less postoperative pain and shorter hospital stays as well [28]. Finally, SV-VATS is associated with reduced risk of short-term postoperative complications, financial burden of patients, and better long-term survival outcome.

Although the obvious advantages of SV-VATS have been recognized, SV-VATS imposes higher requirements on careful patient selection, appropriately experienced anesthetic, and surgical teams, which restrict the more extensive application and further development of SV-VATS to some extent. Currently, SV-VATS can only be performed on selected patients in a few centers. For institution applying this technique, it is important for surgeons and anesthesiologists to select the proper patients in the early phase of learning curve. This is necessary and the first step to decrease the risk of conversion to intubated general anesthesia and complications [29].

Currently, consensus for SV-VATS has been published [30–32]. Surgeons and anesthesiologists can select patients according to the inclusion and exclusion criteria in consensus. However, such consensus is not just for patients with NSCLC, but also other thoracic diseases. Besides, the consensus was based on a larger population of patients, which cannot quantitively predict the risk in an individual patient. For example, patients older than 60 years old are not suggested to undergo SV-VATS in the consensus. However, evidence has showed that patients over 65 years old can still undergo SV-VATS and have a comparable effect with MV-VATS [10, 24]. Besides, BMI > 30 kg/m^2^ is an exclusion criterium of SV-VATS in the consensus. However, research has reported that it was safe and feasible for patients with BMI > 30 kg/m^2^ to undergo SV-VATS and the effect was comparable to MV-VATS [33].

In fact, while making a surgery plan for each patient, clinicians will consciously or subconsciously assign point to each potential risk factors based on the published literature and experience. Plan for treatment will be formed based on the total score in mind. However, such skill is highly subjective and difficult to disseminated to less-experienced surgeons and anesthesiologists. Besides, even expert may make mistakes sometimes. Therefore, a more robust and customized surgery decision model is urgently needed to identify the optimal candidates for SV-VATS among patients with NSCLC.

Nomogram, which has been proven to be capable of assisting the preoperative assessment and surgical planning, is readily used, and interpreted by clinical workers owing to its intuitive features.

In this study, we built an SDS model in the form of nomogram with several clinically and statistically significant predictors via univariate and multivariate logistic regression analysis. Sample size calculations indicated that the minimum requirement for this study is 283, the actual training group sample size is 3,433, which far exceeds the minimum sample size requirement. The inclusion of a significantly larger sample size can enhance the predictive performance of this study and improve the accuracy of the predicted results. Many common and readily accessible information like age, BMI, smoking status, FEV1/FVC, TMN stage, ASA grade and surgical technique are included. Only when the factor is significant in both univariate and multivariate logistic regression analysis can be included in the model and be assigned certain points. Finally, based on the demographic characteristics, oncological information, and operation information from the patient with NSCLC, a total points and probability to perform SV-VATS will be generated from the surgery decision model. Exceptionally, FEV1/FVC was maintained in the model even though it did not reach statistical significance in the multivariate analysis, because it is an important factor that needs to be considered in clinical surgical plan. The computed AUC values of the training group and the testing group were 0.72 and 0.70 respectively, which indicated a high precision in the prediction of the model.

The model is promising in clinical applications, especially in the centers that are new to the technique of SV-VATS. Three major elements are needed for a successful SV-VATS: careful patient selection, appropriately experienced anesthetic, and surgical teams. Although SV-VATS is still a new and challenging technique with a steep learning curve, the latter two elements can be improved by systemic training. Currently, there have been many lobectomy video tutorials based on single-hole the SV-VATS [34]. Besides, the advances in image recognition techniques and artificial intelligence-assisted identification of anatomic sites can also help building a better understanding of a new surgical technique [35]. Our center has also made similar attempts in SV-VATS. the follow-up results are in progress. As for the first element, selecting optimal patients is not just about repeated practice and experience, but it needs a more objective basis. Therefore, with the model we developed, professional and systematic training, and technical improvement, SV-VATS can be developed better and applied more widely in patients with NSCLC.

Nevertheless, several limitations to this study need to be acknowledge. First, the SV-VATS is a relatively new technique and only practicable in a few hospitals by some experienced anesthetists. External validation has not been scheduled, which may reduce the reliability of the SDS model. Second, as a retrospective study, many variables which may strongly influence the surgical options were not able to be collected. Third, there might be potential selection bias due to the limited sample size and surgeon/patient factors. Last, whether the other surgical procedures can be applied to our model remains to be seen and further improvement is needed in future research.

Future endeavors are clearly needed. First, prediction and management of patients who underwent SV-VATS and then converted to general anesthesia. Second, development of surgical learning curve of SV-VATS.

## Conclusion

This SDS model is the first clinical decision-making model developed for an individual NSCLC patient to make decision between SV-VATS and MV-VATS. Age, smoking status, BMI, ACCI, T stage, N stage, FEV1/FVC after inhalation of bronchodilators, ASA grade, surgical technique are important risk factors affecting the chosen of surgical approach. The discovery of risk factors and the construction of SDS model can help surgical team to choose the best surgical approach and answer patient consultations.

### Supplementary Information

**Additional file 1: Supplementary Figure 1.** The procedure of SV-VATS technique.

**Additional file 2.**

## Data Availability

The datasets presented in this article are not readily available because the patients involved in this study are from the Department of Thoracic Surgery of our hospital and are not open to anyone other than the author of this study. Requests to access the datasets should be directed to Runchen Wang, runchen_wang@outlook.com.

## Abbreviations

NSCLC: Non-small cell lung cancer
VATS: Video-assisted thoracoscopic surgery
SV: Spontaneous ventilation
MV: Mechanical ventilation
SDS: Surgical decision-making scoring
FEV1/FVC: Forced expiratory volume in 1 s (FEV1)/forced volume vital capacity (FVC) ratio
BMI: Body mass index
ACCI: Age-adjusted Charlson Comorbidity Index
ASA: American Society of Anesthesiologists
OR: Odds ratio
CI: Confidence intervals
AUC: Area under the receiver operating characteristic curve
DCA: Decision curve analysis
