# Supplementary figures and images for: A surgical Decision-making scoring model for spontaneous ventilation- and mechanical ventilation-video-assisted thoracoscopic surgery in non-small-cell lung cancer patients
Source: BMC Surg. 2023 Sep 25;23:290. doi: 10.1186/s12893-023-02150-z (PMC10519124; doi:10.1186/s12893-023-02150-z)

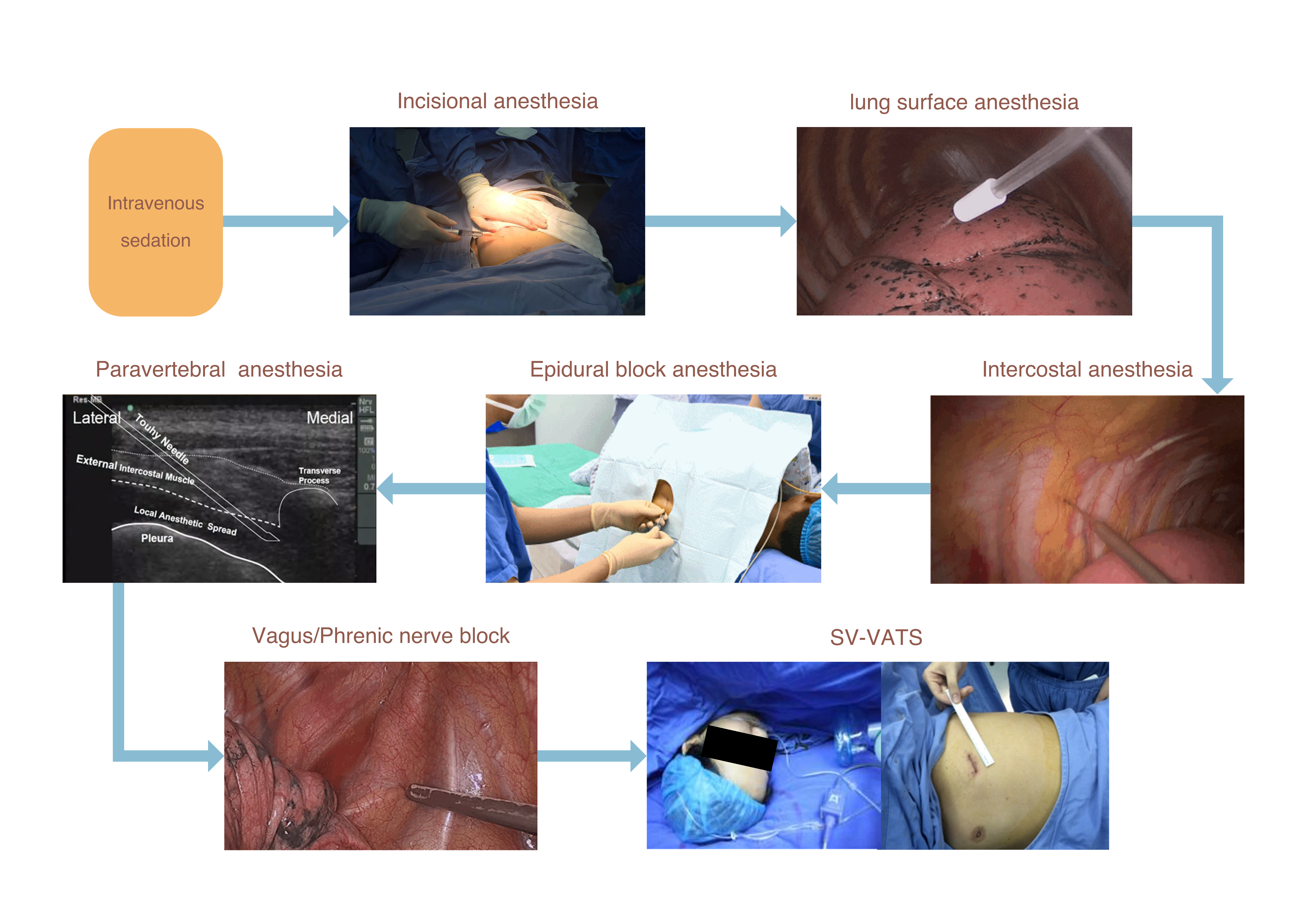

Supplement: Supplementary file 1 — Additional file 1: Supplementary Figure 1. The procedure of SV-VATS technique. [file 12893_2023_2150_MOESM1_ESM.tiff]
