# Supplementary material for: A surgical Decision-making scoring model for spontaneous ventilation- and mechanical ventilation-video-assisted thoracoscopic surgery in non-small-cell lung cancer patients
Source: BMC Surg. 2023 Sep 25;23:290. doi: 10.1186/s12893-023-02150-z (PMC10519124; doi:10.1186/s12893-023-02150-z)
Supplement: Supplementary file 2 — Additional file 2. [file 12893_2023_2150_MOESM2_ESM.docx]

Step1:

Data collection and input: clinicians need to collect following 8 factors from patients: smoking status, BMI, ACCI, T stage, N stage, FEV1/FVC, ASA grade, surgical technique. All these data can be obtained from patient records.

Step2:

Variable transformation and score calculation: each predictive variable has a corresponding scale or axis on the nomogram. For example, for BMI, the scale is from 36-14 kg/m2 with increments of 2 kg/m2. Clinicians and other authors locate the corresponding scale on the nomogram based on the patient’s BMI and convert it into a score. The same method applies to other predictive variables.

Step3:

Score summation and interpretation of predication results: clinicians and other authors sum up the scores of 8 predictive variables to obtain a total score. Then, based on the SDS model on the nomogram, clinicians could find the corresponding total score to determine the probability of the patient undergoing SV-VATS. For example, if the total score is 200, the nomogram indicates a corresponding probability of 15%.

By following these steps and explanations, clinicians can use the nomogram for prediction by inputting patients’ characteristic data, calculating the total score, and interpreting and applying the prediction results based on the SDS model.
